# Spatial gene expression and functional network abnormalities in multiple sclerosis: exploring biological influence on brain functional reorganization

**DOI:** 10.1038/s41398-026-03921-x

**Published:** 2026-03-03

**Authors:** Paolo Preziosa, Matteo Azzimonti, Loredana Storelli, Paola Valsasina, Nicolò Tedone, Monica Margoni, Massimo Filippi, Maria A. Rocca

**Affiliations:** 1 Neuroimaging Research Unit, Division of Neuroscience, IRCCS San Raffaele Scientific Institute, Milan, Italy; 2 Neurology Unit, IRCCS San Raffaele Scientific Institute, Milan, Italy; 3 Vita-Salute San Raffaele University, Milan, Italy; 4 Neurorehabilitation Unit, IRCCS San Raffaele Scientific Institute, Milan, Italy; 5 Neurophysiology Service, IRCCS San Raffaele Scientific Institute, Milan, Italy

**Keywords:** Molecular neuroscience, Predictive markers

## Abstract

In multiple sclerosis (MS), functional network abnormalities arise as structural damage accumulates. However their biological basis and spatial distribution remain unclear. This study investigated the associations between MS-related functional network abnormalities and physiological gene expression using the Allen Human Brain Atlas (AHBA). Five-hundred fifty-eight MS patients and 214 healthy controls (HC) underwent neurological assessment and 3 T MRI; 491 patients also completed a neuropsychological evaluation. Resting-state functional MRI was used to generate degree centrality maps to identify network topography alterations. Spatial correlations between centrality abnormalities (p < 0.01 uncorrected) and the expression of 3634 MS-related genes was evaluated using AHBA and the Multimodal Environment for Neuroimaging and Genomic Analysis. Genes showing significant associations (p < 0.001, R^2^ ≥ 0.15) underwent pathway enrichment analysis (p < 0.05, Bonferroni-corrected). Compared to HC, MS patients showed higher centrality mainly in the default-mode network (DMN), linked to genes regulating inflammation resolution and immune functions, and lower centrality in regions mostly located in the salience network and cerebellum, associated with genes implicated in cytokine response. Compared to HC and relapsing-remitting MS, progressive MS patients showed higher centrality in DMN and cerebellar regions, correlating with genes related to epigenetic and mitochondrial functions. Of the MS cohort, 144 (29.3%) patients were cognitively impaired. Compared to cognitively preserved MS and HC, they showed higher centrality in DMN and mesial temporal lobe regions, negatively correlated with expression of *DNASE1*, regulating DNA degradation, and *CP*, encoding ceruloplasmin, involved in iron homeostasis and potentially iron-driven neurodegeneration. Physiological regional gene expression spatially correlates with MS-related functional network alterations. Biological factors may shape regional vulnerability or resilience to MS pathology, influencing functional reorganization.

## Introduction

Multiple sclerosis (MS) is a chronic inflammatory, demyelinating and neurodegenerative disease of the central nervous system (CNS) characterized by a gradual accumulation of pathological abnormalities from its biological onset [1, 2]. Currently, the correlation between in-vivo structural MRI measures and clinical manifestations remains suboptimal [3]. One possible explanation is the variable functional brain plasticity and network reorganization across different disease stages, which can be explored using functional MRI [4, 5]. In the early phases, increased resting-state (RS) functional connectivity (FC) may reflect a compensatory mechanism [4, 5]. As the disease progresses, these patterns become more heterogeneous and potentially maladaptive [4, 5] contributing to differentiate clinical phenotypes [6, 7], explain more severe disability [8–13], and cognitive impairment [14–18].

Recent advancements in neuroimaging analysis have expanded RS FC analyses beyond classical large-scale resting-state networks (RSNs) [4, 5]. The application of graph theory in MS research allows computation of network metrics that assess local and global connectivity properties [19–25]. Among these, degree centrality quantifies the relative importance of each node within networks based on its connections, providing a measure of “hubness” [26]. Voxel-wise analyses of centrality in MS have revealed widespread network alterations. Lower centrality in the sensorimotor network (SMN) has been linked to more severe physical disability and a progressive disease phenotype [25, 27], whereas lower centrality in the salience network (SN) has been associated with cognitive impairment [25]. These findings suggest that the loss of functional connections may isolate key brain regions, contributing to clinical manifestations, likely driven by structural damage accumulation. This is supported by the earlier involvement of structural networks compared to functional ones [20] and the significant associations of functional abnormalities with brain lesion volume and atrophy [25]. Conversely, the default-mode network (DMN) has consistently shown increased centrality, which correlates with cognitive impairment [25, 28, 29]. It has been hypothesized that, in the presence of widespread structural damage, FC becomes more confined to a limited number of dominant activity patterns centered around network hubs. Over time, this may lead to hub overload and, ultimately, network collapse [5, 7, 30, 31].

Despite these insights, the biological mechanisms driving these clinically relevant RS FC modifications and the factors influencing their spatial distribution remain poorly understood. Advances in high-throughput gene expression analysis have enabled the construction of brain-wide transcriptomic atlases, such as the Allen Human Brain Atlas (AHBA) [32], allowing for transcriptional profiling of *post-mortem* brain tissue with high spatial resolution. These atlases provide a valuable resource for investigating the molecular mechanisms underlying disease-related abnormalities [33]. Spatial correlation analyses between disease-specific regional brain abnormalities and regional physiological gene expression patterns may help identify molecular determinants of brain regions’ vulnerability or resilience to pathology [34]. This approach has been successfully employed to explain characteristic patterns of gray matter (GM) atrophy in MS and other neuro-inflammatory disorders [35–37]. A similar framework can be leveraged to improve our understanding of RSN reorganization in these conditions.

Given this background, we investigated whether the regional expression of specific genes in the healthy brain is associated with RSN reorganization in MS. Using a large, well-characterized cohort of MS patients, we performed spatial cross-correlation analyses between regional brain-wide gene expression profiles from the AHBA and degree centrality abnormalities in MS patients compared to healthy controls (HC). Additionally, we explored the relationship between gene expression patterns and FC abnormalities in specific disease subgroups stratified by disease clinical phenotype and cognitive status.

## Materials and methods

### Study design and population

This retrospective observational study was approved by the Institutional Ethical Standards Committee on Human Experimentation at IRCCS Ospedale San Raffaele (Protocol N° 2013-33). Written informed consent was obtained from all participants in accordance with the Declaration of Helsinki.

From the database of the Neuroimaging Research Unit, IRCCS San Raffaele Scientific Institute (Milan, Italy), we selected 558 consecutive MS patients and 214 HC who underwent the same neuropsychological and MRI protocols. MS patients were eligible for inclusion if they were at least 18 years old, right-handed (defined as Edinburgh Handedness Inventory >50 [38]), and had a diagnosis of MS according to the 2017 revised McDonald criteria [39]. Additional inclusion criteria required that MS patients be relapse- and steroid-free for at least three months prior to MRI, have no significant neurological (other than MS) or psychiatric conditions, no history of drug or alcohol abuse, and be on stable disease-modifying treatment (DMT) for at least three months. HC had to be right-handed, have no neurologic diseases or systemic disorders affecting the CNS, and have a completely normal neurologic examination.

### Clinical and neuropsychological assessment

Within 3 days of MRI acquisition, all MS patients underwent a comprehensive neurological examination, with rating of the Expanded Disability Status Scale (EDSS) score [40], recording of current DMT, and clinical phenotype classification (i.e., relapsing-remitting [RR] or progressive [P] MS).

Neuropsychological evaluation was conducted using the Brief Repeatable Battery of Neuropsychological Tests (BRB-N), version A, administered by an experienced neuropsychologist [41]. *Z*-scores for all BRB-N tests were calculated by correcting raw scores for age, sex, and education according to updated Italian normative data [42]. Test failure was defined as a score at least 1.5 standard deviations below normative values. Impairment in a single domain was defined as failure in at least one test assessing that domain, whereas cognitive impairment was defined as failure in at least two cognitive domains [43]. Patients who were unable to complete the BRB-N due to excessive disability or voluntarily withdrawal (n = 67) were excluded from the analysis of cognitive data.

### MRI acquisition

The MRI protocol was performed using two 3.0 T scanners (Scanner 1: Achieva, n = 334 MS patients/110 HC; Scanner 2: Ingenia, n = 224 MS patients/104 HC; Philips Medical Systems, Eindhoven, The Netherlands) and included the following sequences: (1) T2*-weighted echo planar imaging sequence for RS functional MRI (fMRI); (2) dual-echo turbo spin echo (Scanner 1) or variable flip angle three-dimensional (3D) T2-weighted fluid-attenuated inversion recovery (FLAIR) (Scanner 2) for white matter (WM) lesion volume assessment; (3) 3D T1-weighted fast field echo for brain atrophy analysis. During RS fMRI acquisition, participants were instructed to keep their eyes closed, to remain motionless, and not to focus on any particular thought. All participants ensured that they had not fallen asleep during the scanning, according to a questionnaire delivered after the MRI session. Detailed MRI parameters for each scanner are provided in the supplementary material.

### Structural MRI analysis

For Scanner 1, focal WM lesions were manually contoured on dual-echo scans using a semiautomatic local thresholding segmentation technique (Jim 7.0, Xinapse Systems Ltd, Colchester, UK). For Scanner 2, brain T2-hyperintense WM lesions were identified and segmented by a fully automated approach based on a cascade of two 3D patch-wise convolutional neural networks, using 3D FLAIR and 3D T1-weighted MRI sequences as input images [44]. Total brain T2-hyperintense WM lesion volume (T2-LV) was calculated from lesion mask after visual inspection of the results. After T1-hypointense WM lesions refilling, normalized brain (NBV), cortical (NcGMV), deep GM (NDGMV) and WM (NWMV) volumes were obtained using FSL SIENAX2 [45] for acquisitions from both Scanners. DGMV was defined as the sum of normalized volumes of the thalamus, caudate, putamen, pallidum, amygdala and nucleus accumbens.

### Degree centrality analysis

After fMRI data preprocessing (detailed in the supplementary material), we calculated voxel-wise centrality maps. Degree centrality, hereafter referred to as centrality, represents the total number of connections of a given voxel with any other GM voxel. To exclude from analysis voxels in WM areas or those without a reliable RS fMRI signal, RS fMRI images were masked using the corresponding GM masks obtained by thresholding a standard GM probability atlas in the MNI space (available in SPM12 software) at 0.20. Binary centrality maps were calculated using the “REST-DC” toolkit embedded in the REST V1.8 package (restfmri.net/forum/REST_V1.8), as previously described [25]. Centrality of each brain voxel was measured using voxel-wise Pearson correlation analysis with all other GM voxels, considering only positive correlation coefficients higher than 0.25 to avoid weak correlations and spurious contributions [46]. Finally, centrality maps were converted through Fisher Z-transformation to account for individual variability [26].

### Statistical analyses

Demographic and clinical variables were compared between MS patients and HC, as well as between MS subgroups based on clinical phenotypes (RRMS or PMS) and cognitive status (preserved or impaired), using the Chi-square or Mann-Whitney test as appropriate. MRI features were compared using sex-, age- and scanner-adjusted linear models. T2-LV was log-transformed before analysis for normalization. A p-value < 0.05 was considered statistically significant. All computations were performed using SPSS (IBM® SPSS® Statistics, version 26.0).

Using a general linear model and the theory of Gaussian fields, voxel-wise between-group comparisons of centrality were performed using SPM12 and full factorial models, including age, sex and scanner as covariates. Comparisons were made between all MS patients and HC, as well as between MS subgroups. To increase specificity in the associations with gene expression, analyses were restricted to the following contrasts: (a) all MS patients versus HC; (b) RRMS patients versus PMS and HC (conjunction analysis) [47]; (c) PMS patients versus RRMS and HC (conjunction analysis); (d) cognitively preserved MS patients versus cognitively impaired MS and HC (conjunction analysis); and (e) cognitively impaired MS patients versus cognitively preserved MS and HC (conjunction analysis). Results were all assessed at p < 0.001 uncorrected (cluster extent k = 20).

Second-level maps derived from centrality between-group comparisons were used as inputs for the analysis of spatial association with gene expression data. In line with the hierarchical general linear modelling framework [48] and with previous works using similar methodology [37, 49, 50], input maps underwent a light thresholding at p < 0.01 (uncorrected), to achieve a good trade-off between the need for data cleaning and adequate sampling of voxels for higher-level statistics.

### Spatial associations between centrality abnormalities and gene expression

Brain-wide gene expression profiles were obtained from microarray data in the AHBA, which comprises transcriptomic data for over 20000 genes taken from approximately 3700 spatially distinct tissue samples from six adult neurotypical brains (aged 24–57 years). Data are publicly available from the Allen brain institute (http://human.brain-map.org) [32].

For our analysis, a selection of genes associated with MS was included, selected using the OpenTarget Platform (https://platform.opentargets.org/, access date: January 15^th^, 2025) with the search term “Multiple Sclerosis”. We obtained a list of 3634 genes associated with the disease. Spatial associations between imaging maps (t-value maps from statistical comparisons of centrality values) and selected gene expressions were explored using the Multimodal Environment for Neuroimaging and Genomic Analysis (MENGA) platform [51] following published guidelines [52], as previously done [35].

Transcriptomic data were obtained from a processed version of the Allen human brain database. Pre-processing of the transcriptomic data from available atlases was carried out to reduce variability and ensure consistency and reproducibility [52]. This process included:

(i) Representative probe selection: For each gene transcript, only one probe was selected to represent its expression across all donors. Since 71% of genes in the AHBA were measured with multiple probes, the representative probe for each gene was selected based on the distribution of expression values (most symmetric and least skewed distribution) to minimize non-linearity effects in microarray measurements [51].
(ii) Normalization of the expression measure: To mitigate donor-specific effects, normalization was applied to each gene’s expression for each donor separately to reflect its relative expression across brain regions. Specifically, z-score normalization was employed:
  $${z}_{{score}}=\frac{{x}_{i}-\,\bar{x}}{{\rm{\sigma }}}$$
  where ${x}_{i}$ is the expression value of a specific gene in a single sample, $\bar{x}$ is the mean, and ${\rm{\sigma }}$ is the standard deviation of expression values for that donor.

Image maps in MNI ICBM152 space were resampled to match AHBA coordinates separately for each donor to ensure image-to-sample spatial correspondence, using MNI coordinates of each sample provided by AHBA. Image data were normalized to z-scores and each image sample was estimated as the average of the voxels within a 3D window of a specified size (here set to 5 mm) centred on the MNI coordinates of the microarray sample. Since only two of the six donors were sampled across the whole brain, our analyses were restricted to the left hemisphere of the brain to increase the robustness of the results.

Spatial associations between imaging and genomic data were assessed using weighted multiple regression within the MENGA platform. To avoid possible overfitting of the regression model, since the six AHBA donors carry similar information in terms of gene expression, a principal component analysis was first applied to the gene expression data to extract the principal components explaining at least 95% of the total variance in the data, which were then used as regressors in the analysis. The resulting multivariate cross-correlation coefficient (R^2^-adjusted) represented the proportion of the total image variability explained by genomic data. The information on the directionality of the imaging-genomic data correlation was also given.

A bootstrapping approach, resampling genomic data 1000 times, was employed to estimate the reliability of the cross-correlation. The chance likelihood was calculated as [51]:

$${chanche}\,{likelihood}=\frac{{number}\,{of}\,{instances}({R}^{2}\, > \,{R}_{{or}}^{2})}{{number}\,{of}\,{bootstraps}}$$

Where ${R}_{{or}}^{2}$ is the value of the coefficient obtained from real data, and ${R}^{2}$ is that obtained using the bootstrapped genomic data. It returned the probability that genomic data be unrelated to image values. Thus, smaller values of chance likelihood denoted a higher reliability of the obtained results.

Finally, an auto-correlation analysis on genomic data is also given by calculating Pearson’s correlation coefficient of the messenger ribonucleic acid (mRNA) data for each pair of AHBA donors, returning information about the biological variability of the mRNA expression across the six sampled brains.

### Enrichment analysis

Genes with significant and reliable spatial associations (chance likelihood <0.001 and auto-correlation ≥0.2) and a cross-correlation with a R^2^ ≥ 0.15 in relation to the t value map of centrality alterations were analyzed for overrepresented biological processes, cellular components, and molecular functions using the ToppGene Suite (http://toppgene.cchmc.org) [35]. This is a free and open-access portal for functional enrichment analysis of gene lists [53], allowing to better explore genetic functionalities. ToppGene Suite uses 14 annotation categories, including Gene Ontology (GO) terms, to generate a representative profile of the input genes and identify over-representative terms. Hypergeometric distribution with Bonferroni correction (p < 0.05) was implemented to assess statistical significance. For this study, the following GO terms were explored: “molecular function”, corresponding to activities performed by individual gene products, “biological process”, constituting larger processes accomplished by multiple molecular activities, and “cellular component”, representing the location(s), relative to cellular structures, where a gene product performs its function.

### Brain cell type specific gene expression

To investigate whether the expression of significant genes was associated with specific CNS cell type, gene expression was analyzed using the open-source R/Shiny tool available at http://celltypes.org/brain [54]. We selected the *Darmanis* database of human brain transcriptome at single cell level [55]. Briefly, the tool started from published raw data on human transcriptome, which are reprocessed using a standardized pipeline for sequencing alignment and gene expression normalization [54]. Then, cell type-associated gene expression was measured considering three types of gene-wise cell-type-relative expression measurements: specificity, enrichment, and absolute expression levels. For each cell type, mRNA expression data were normalized and presented using the quantile method [56].

## Results

### Demographic, clinical, neuropsychological and structural MRI analysis

Compared to HC, MS patients were significantly older (p = 0.008), had fewer years of education (p < 0.001), higher brain T2-LV, as well as lower NBV, NcGMV, NDGMV and NWMV (p < 0.001) (Table 1). Sex distribution did not differ significantly between groups (p = 0.073) (Table 1).

**Table 1:** Main demographic, clinical and structural MRI characteristics of HC and MS patients and of MS patients according to their clinical phenotype and the presence of cognitive impairment.

	HC (n = 214)	MS (n = 558)	p	Clinical phenotype		p	Cognitive impairment		p
				RRMS (n = 365)	PMS (n = 193)		Cognitively preserved MS (n = 347)	Cognitively impaired MS (n = 144)	
Median age (IQR) [years]	38.4 (27.4;50.9)	42.4 (33.3;50.6)	0.008	37.9 (29.3;45.2)	49.9 (43.7;57.7)	<0.001	41.8 (31.2;48.8)	44.9 (36.7;54.4)	0.002
Sex: Female/Male (%)	116/98 (54/46)	342/216 (61/39)	0.073	230/135 (63/37)	112/81 (58/42)	0.250	214/133 (62/38)	79/65 (55/45)	0.161
Median education (IQR) [years]	16.0 (13.0;18.0)	13.0 (13.0;16.0)	<0.001	13.0 (13.0;17.0)	13.0 (10.0;13.0)	<0.001	13.0 (13.0;17.0)	13.0 (10.0;13.0)	<0.001
Median disease duration (IQR) [years]	-	11.5 (4.1;18.0)	-	8.0 (2.5;14.0)	17.7 (12.0;23.4)	<0.001	10.1 (3.4;17.6)	14.4 (6.3;21.6)	<0.001
Clinical phenotype (%)	-	365 (65) RR; 193 (35) P	-	365 (100) RR	133 (69) SP; 60 (31) PP	-	245 (71) RR; 69 (20) SP; 33 (9) PP	62 (43) RR; 58 (40) SP; 24 (17) PP	<0.001
Median EDSS (IQR)	-	2.5 (1.5;5.5)	-	2.0 (1.5;3.5)	6.0 (5.0;7.0)	<0.001	2.0 (1.0;4.5)	4.5 (2.5;6.0)	<0.001
DMT*: None/MET/HET (%)	-	143/276/139 (26/49/25)	-	63/211/91 (17/58/25)	80/65/48 (41/34/25)	<0.001	86/169/92 (25/49/26)	45/63/36 (31/44/25)	0.331
Scanner (%) [Achieva/Ingenia]	110/104 (51/49)	334/224 (60/40)	0.033	228/137 (62/38)	106/87 (55/45)	0.084	187/160 (54/46)	86/58 (60/40)	0.236
Median brain T2-LV^#^ (IQR) [ml]	0.00 (0.00;0.14)	4.01 (1.52;9.91)	<0.001	2.92 (1.12;6.80)	8.54 (3.34;18.64)	<0.001	3.30 (1.27;6.90)	10.61 (3.69;20.48)	<0.001
EM NBV (SE) [ml]	1581 (4)	1532 (3)	<0.001	1543 (4)	1504 (5.3)	<0.001	1545 (3)	1496 (5)	<0.001
EM NcGMV (SE) [ml]	640 (3)	614 (3)	<0.001	617 (3)	602 (3.7)	0.001	616 (2)	593 (4)	<0.001
EM NDGMV (SE) [ml]	55.3 (0.3)	51.0 (0.2)	<0.001	52.2 (0.3)	48.5 (0.4)	<0.001	52.2 (0.3)	47.7 (0.4)	<0.001
EM NWMV (SE) [ml]	745 (3)	722 (2)	<0.001	728 (2)	711 (3.4)	<0.001	734 (2)	710 (3)	<0.001

Comparisons performed using Chi square test (sex, scanner, clinical phenotype, DMT) and Mann-Whitney U test (age, education, disease duration, EDSS). Sex-, age- and scanner-adjusted linear models were applied for MRI features.

^*^MET: interferon beta, glatiramer acetate, teriflunomide, dimethyl fumarate; HET: fingolimod, siponimod, cladribine, natalizumab, ocrelizumab.

^#^comparison performed on log scale.

*DMT* disease modifying treatment, *EM* estimated mean, *EDSS* expanded disability status scale, *HC* healthy controls, *HET* high efficacy treatment, *IQR* interquartile range, *MET* moderate efficacy treatment; *MS* multiple sclerosis, *ml* millilitre, *NBV* normalized brain volume, *NcGMV* normalized cortical gray matter volume, *NDGMV* normalized deep gray matter volume, *NWMV* normalized white matter volume, *P* progressive, *PP* primary progressive, *RR* relapsing-remitting, *SE* standard error, *SP* secondary progressive, T2-LV = T2-hyperintense WM lesion volume.

Among MS patients, 365 (65%) had RRMS and 193 (35%) had PMS. The significant age difference observed between the entire MS group and HC was primarily driven by PMS patients, who were significantly older than RRMS patients (p < 0.001). Moreover, compared to RRMS, PMS patients had fewer years of education, longer disease duration, higher EDSS score, and were less frequently treated with DMTs (p < 0.001). Structural MRI analysis showed higher brain T2-LV as well as lower NBV, NcGMV, NDGMV and NWMV (p ≤ 0.001) in PMS compared to RRMS patients (Table 1).

Of the 558 MS patients, 491 (88%) completed the BRB-N. Among them, 144 (29%) were classified as cognitively impaired. The most frequently impaired cognitive domains among cognitively impaired MS patients were information processing speed/attention (88%) and verbal memory (82%) (see Supplementary Table 1 for details).

Compared to cognitively preserved MS patients, those with cognitive impairment were significantly older, had fewer years of education, longer disease duration and higher EDSS score (p ≤ 0.002). They also had significantly higher brain T2-LV and as well as lower NBV, NcGMV, NDGMV and NWMV (p < 0.001) (Table 1).

### Patterns of degree centrality abnormalities, spatial correlations with gene expression and enrichment analysis

#### MS patients versus HC

**Fig. 1:**
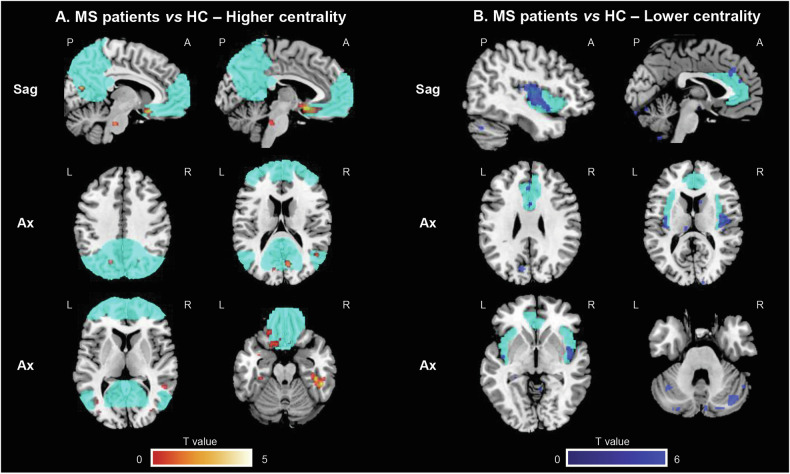
SPM12 analysis showing voxel-wise differences in centrality between MS patients and HC. Clusters of higher centrality in MS patients are shown in red-yellow scale (**A**), while clusters of lower centrality are shown in blue-light blue scale (**B**); images are thresholded at p < 0.001, uncorrected, cluster extent k = 20. Results are superimposed on the MNI152 atlas and overlaid on a standard template mask of the default mode network (cyan) for higher centrality, and on a standard template of the salience network (cyan) for lower centrality. Images are presented in neurological convention. A anterior; Ax axial; HC healthy controls; L left; MS multiple sclerosis; R right; P posterior; Sag sagittal; vs versus.

**Fig. 2:**
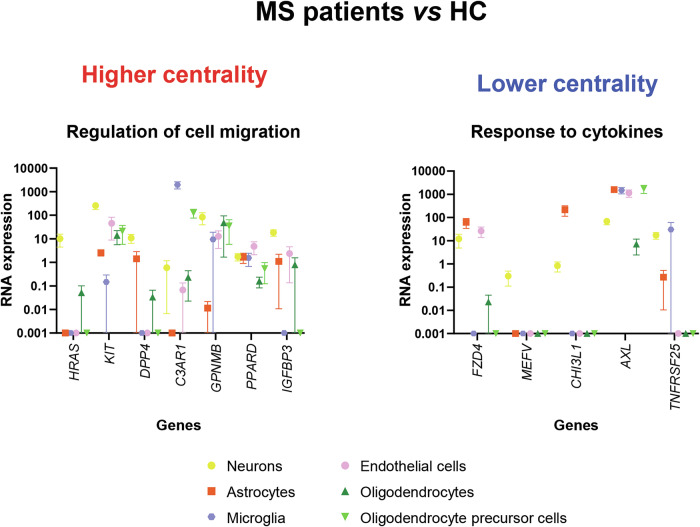
Overview of specific RNA expression in brain cell types of the different genes showing a significant and reliable spatial association (chance likelihood <0.001 and auto-correlation ≥0.2) and an adjusted cross-correlation with a R^2^ ≥ 0.15 with the voxel-wise differences in centrality between MS patients and HC and that were found to be enriched at enrichment analyses. RNA expression of the different genes has been obtained from McKenzie et al. [54] using gene expression derived from Darmanis brain single cell human atlas transcriptome [55]. Error bars represent the standard error of the mean. RNA expression data have been quantile normalized, and are expressed on a logarithmic scale; constant value of 0.001 has been added to all data points to allow representation of zero values. See text for further details. HC healthy controls; MS multiple sclerosis; RNA ribonucleic acid; vs versus.

Compared to HC, MS patients showed significantly higher centrality located in the bilateral precuneus, bilateral orbitofrontal and inferior temporal cortices, regions known to be part of the DMN (p < 0.001, uncorrected) (Fig. 1). This pattern showed significant spatial correlation with the expression of 17 genes (p < 0.001) (Supplementary Table 2). Enrichment analysis showed overrepresentation of molecular pathways related to calcium-signalling, and biological processes involved in cell migration and immune system function (Table 2). Notably, genes associated with cell migration are mainly expressed in neurons, microglia and endothelial cells, and are involved in different CNS and immune system functions, including resolution of inflammation and damage repair (Fig. 2).

**Table 2:** Enrichment analysis showing significantly overexpressed pathways (molecular function, biological processes, cellular components) among genes showing spatial association with centrality abnormalities in MS patients, also according to clinical phenotype.

Centrality abnormalities	GO terms	ID	Name	p	Bonferroni	N of genes
Higher centrality in MS vs HC	Molecular function	GO:0004683	Calmodulin-dependent protein kinase activity	<0.001	0.036	2
	Biological process	GO:0060374	Mast cell differentiation	<0.001	0.023	2
		GO:0030334	Regulation of cell migration	<0.001	0.046	7
Lower centrality in MS vs HC	Molecular function	GO:0034097	Response to cytokine	<0.001	0.046	5
Higher centrality in PMS vs RRMS and HC	Molecular function	GO:0003682	Chromatin binding	<0.001	0.014	5
		GO:0008327	Methyl-CpG binding	<0.001	0.028	2
		GO:0043021	Ribonucleoprotein complex binding	<0.001	0.048	3
	Biological process	GO:0040029	Epigenetic regulation of gene expression	<0.001	0.028	4
		GO:0006338	Chromatin remodelling	<0.001	0.045	5
	Cellular component	GO:0045120	Pronucleus	<0.001	0.014	2
		GO:0005721	Pericentric heterochromatin	<0.001	0.019	2
		GO:0005759	Mitochondrial matrix	<0.001	0.029	4

*HC* healthy controls, *GO* gene ontology, *MS* multiple sclerosis, *RRMS* relapsing-remitting multiple sclerosis, *PMS* progressive multiple sclerosis.

MS patients also showed significantly lower centrality compared to HC in the bilateral insula (a key region of the SN), as well as in the left and right cerebellum (crus I, lobules VI-IX) (p < 0.001, uncorrected) (Fig. 1). This pattern of reduced centrality was spatially correlated with the expression of 10 genes (p < 0.001) (Supplementary Table 3). These genes were significantly enriched for processes related to cellular response to cytokines (p = 0.046, Bonferroni corrected), suggesting that a higher expression of pro-inflammatory cytokines is associated with a reduced centrality observed in these regions. They are mainly expressed in astrocytes, endothelial cells, and neurons (Fig. 2).

#### MS clinical phenotypes

**Fig. 3:**
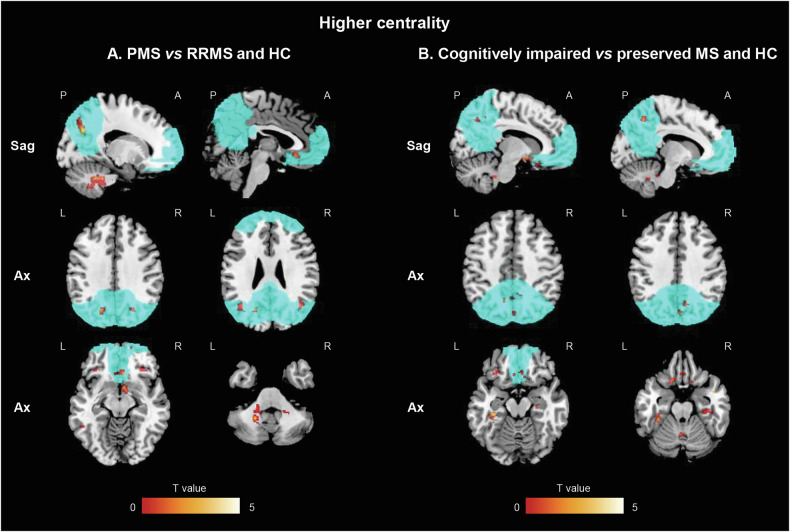
SPM12 analysis showing voxel-wise differences in centrality between MS patients, according to their clinical phenotype and cognitive status, and HC. **A** Higher centrality in PPMS vs RRMS and HC; (**B**) Higher centrality in cognitively impaired MS patients vs cognitively preserved and HC. Clusters of higher centrality are shown in red-yellow scale; images are thresholded at p < 0.001, uncorrected, cluster extent k = 20. Results are superimposed on the MNI152 atlas and overlaid on a standard template mask of the default mode network (cyan). Images are presented in neurological convention. A anterior; Ax axial; HC healthy controls; L left; MS multiple sclerosis; R right; P posterior; PMS progressive MS; RRMS relapsing-remitting MS; Sag=sagittal; vs versus.

**Fig. 4:**
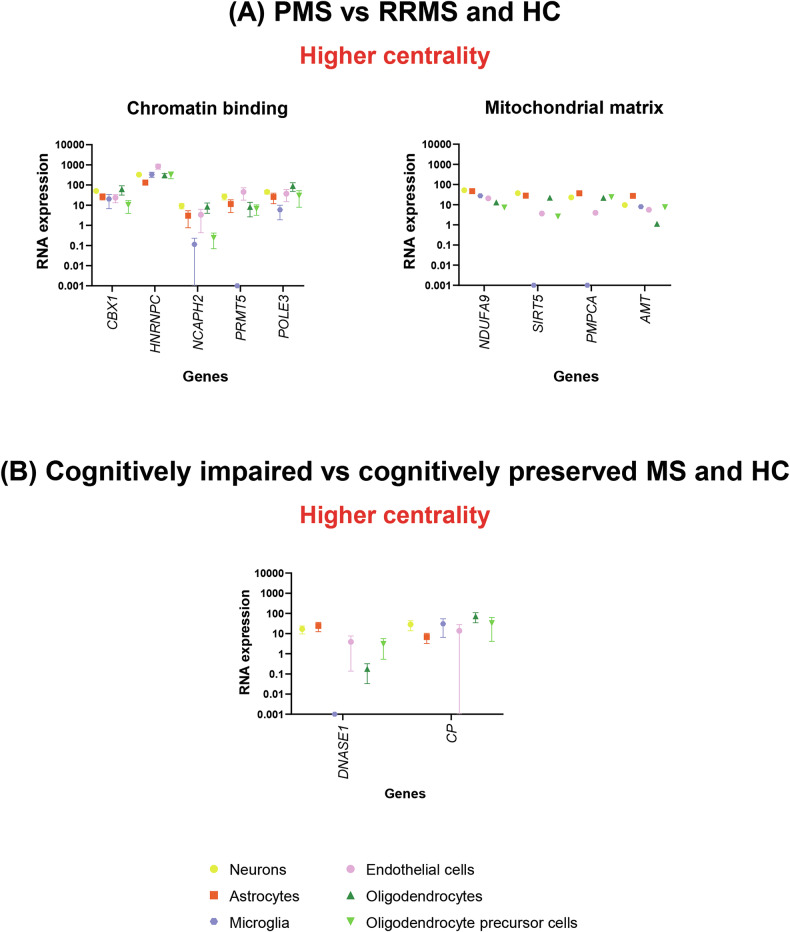
Overview of specific RNA expression in brain cell types of the different genes showing a significant and reliable spatial association (chance likelihood <0.001 and auto-correlation ≥0.2) and an adjusted cross-correlation with a R^2^ ≥ 0.15 with the voxel-wise differences in centrality between MS patients, according to their clinical phenotype and cognitive status, and HC. **A** genes showing spatial association with maps of higher centrality in PMS vs RRMS and HC, which were found to be enriched at enrichment analyses; (**B**) genes showing spatial association with maps of higher centrality in cognitively impaired MS vs preserved and HC. RNA expression of the different genes has been obtained from McKenzie et al. [54] using gene expression derived from Darmanis brain single cell human atlas transcriptome [55]. Error bars represent the standard error of the mean. RNA expression data have been quantile normalized, and are expressed on a logarithmic scale; constant value of 0.001 has been added to all data points to allow representation of zero values. See text for further details. HC healthy controls; MS multiple sclerosis; PMS progressive multiple sclerosis; RNA ribonucleic acid; RRMS relapsing-remitting multiple sclerosis; vs versus.

Compared to RRMS patients and HC, PMS patients showed significantly higher centrality in the precuneus and angular gyrus (known to be part of the DMN), but also in the right and left cerebellum (lobules VI-VIII-IX) and orbitofrontal cortex (p < 0.001, uncorrected) (Fig. 3). This pattern was significantly correlated with the physiological expression of 14 genes (p < 0.001) (Supplementary Table 4). Enrichment analysis revealed significant over-representation of epigenetic regulation pathways. Moreover, four of these genes encoded proteins localized in the mitochondrial matrix (p = 0.029, Bonferroni corrected), suggesting their involvement in mitochondrial energy production. All these genes were found to have ubiquitous expression across CNS cell types (Fig. 4).

PMS patients also showed reduced centrality in bilateral insula and anterior cingulate cortex, as well as the thalamus bilaterally and the right caudate nucleus (p < 0.001, uncorrected). This spatial map showed no significant spatial associations with transcriptomic data (data not shown).

RRMS patients showed no significant suprathreshold cluster in the conjunction analysis compared to PMS patients and HC.

#### Cognition

Compared to cognitively preserved MS patients and HC, cognitively impaired MS patients showed significantly higher centrality in bilateral mesial temporal lobe regions, the right and left precuneus (part of the DMN) and the orbitofrontal cortex (p < 0.001, uncorrected) (Fig. 3). This pattern was negatively associated with the expression of 2 genes, *DNASE1* and *CP* (p < 0.001) (Supplementary Table 5). *DNASE1* encodes for an enzyme involved in desoxyribonucleic acid (DNA) degradation, which may play a role in neuronal apoptosis. It is predominantly expressed in neurons and astrocytes (Fig. 4). *CP* encodes for ceruloplasmin, a protein involved in iron homeostasis in the CNS and ubiquitously expressed among CNS cell types (Fig. 4).

Cognitively impaired MS also showed small clusters of lower centrality in left and right thalamus, left insula, left precentral gyrus and right postcentral gyrus (p < 0.001, uncorrected). This spatial map showed no significant spatial associations with transcriptomic data (data not shown).

Cognitively preserved MS patients exhibited no significant suprathreshold centrality clusters compared to the other groups in the conjunction analysis.

## Discussion

This retrospective cross-sectional study investigated the spatial correlations between RSN abnormalities in MS, measured through degree centrality, and regional gene expression profiles derived from the AHBA transcriptomic data. MS patients had increased centrality in areas mainly of the DMN and decreased centrality in regions of the SN and cerebellum compared with HC. These abnormalities were associated with the expression of genes involved in inflammation resolution, immune function and cytokine signalling. Notably, increased centrality in regions of the DMN was more pronounced in PMS patients and correlated with the expression of genes regulating epigenetic and mitochondrial function. Additionally, cognitively impaired MS patients showed higher centrality in DMN and mesial temporal lobe regions, negatively correlated with *DNASE1* and *CP* expression.

Our structural MRI findings align with previous studies, revealing a significant accumulation of structural damage in MS patients, in terms of higher brain T2-LV and lower NBV, NcGMV, NDGMV, and NWMV compared to HC, being most pronounced in PMS [57–59] and in patients with cognitive impairment [41, 60–63].

As previously described [25, 28, 29], MS patients showed higher centrality in regions of the DMN compared to HC. This likely reflects widespread MS-related structural damage accumulation that reduces brain flexibility in functional interactions and reinforces hub-centered activity [25, 30, 64]. These regions with higher centrality were also characterized by the physiological expression of genes involved cell migration regulation. Among these, we found higher expression of genes implied in inflammation resolution and damage repair, such as *DPP4* [65], *HRAS* [66], *GPNMB* [67, 68] and *IGFBP3* [69], and lower expression of genes involved in immune cell recruitment, such as *C3AR1* [70]. This may indicate that these regions have stronger anti-inflammatory and pro-reparative activities. A higher expression of genes that limit inflammation and promote neuroprotection may prevent structural damage accumulation, including demyelination, but also neuro-axonal and synaptic loss. Furthermore, a lower expression of genes involved in innate immune cell activation [71] suggests these brain areas may be inherently more resistant to MS-related damage. This potential resilience may confer structural and functional protection against disconnection, thus contributing to the crucial role of DMN hubs in the functional connectome. These genes are mainly expressed in neurons and endothelial cells, key components of the neurovascular unit that maintains brain energy homeostasis [72]. Neurovascular unit dysfunction has been implicated in MS pathology [73] and our findings are particularly relevant because the fMRI blood-oxygen-level-dependent (BOLD) signal depends on the increase in blood flow and in the ratio between oxy- and deoxyhemoglobin during neuronal activation to meet higher energy demands [74]. Brain regions with stronger neurovascular resilience to MS-related damage, potentially conferred by higher expression of neuroprotective genes, might better regulate their energy supply and preserve centrality despite structural damage.

MS patients also showed lower centrality in key SN regions, including the insular cortex [25], as well as in the cerebellum [25, 75] compared to HC. This pattern correlated with the physiological expression of genes involved in cytokine signalling and predominantly expressed in astrocytes, neurons, and endothelial cells. In MS, pro-inflammatory cytokines have detrimental effects on resident CNS cells [76, 77], and this could be particularly pronounced in regions with higher cytokine sensitivity, as indicated by their elevated gene expression in the healthy brain. These findings suggest that higher cytokine signaling gene expression in the insula and cerebellum may contribute to the observed lower centrality in MS patients. Additionally, this could help explain why these regions are particularly prone to developing focal GM lesions, as shown in pathological studies [78, 79]. WM damage affecting long-range connections could also contribute to the reduced centrality observed in these regions [25].

When looking at disease phenotypes, compared to RRMS patients and HC, PMS patients showed significantly higher centrality in DMN regions [25, 80]. This finding likely reflects more extensive structural damage, which intensifies maladaptive network reorganization around brain hubs [5, 7, 30, 31]. This pattern of higher centrality correlated with the expression of genes involved in epigenetic regulation and primarily localized in the nucleus. Epigenetic modulation in these regions may allow for greater adaptability to environmental stressor and contribute to the ability to modulate gene expression in response to pathological stimuli [81–83]. Moreover, these regions showed higher expression of genes located in the mitochondrial matrix, which likely reflects the high energy demands of brain hubs, given the costly nature of their long-range neural connections [84]. Higher mitochondrial energy production capacity may also contribute to their relative resilience to MS-related damage. Importantly, these genes were ubiquitously expressed across CNS cell types, highlighting their fundamental role in cellular homeostasis [85].

PMS patients also showed lower centrality in SN regions and deep GM nuclei compared to HC and RRMS patients, as previously observed [6, 25]. However, no significant cross-correlations with transcriptomic data were found, possibly due to the heterogeneity of pathological mechanisms in PMS [1, 86].

Compared to cognitively preserved MS patients and HC, cognitively impaired MS patients showed higher centrality in the DMN, as well as in bilateral mesial temporal lobe and orbitofrontal cortex, regions where higher RS FC has been consistently associated with cognitive impairment [13, 15, 25, 30, 87]. This rigid hub-centered connectivity aligns with reduced cognitive efficiency and network overload [30, 88]. This pattern of higher centrality was spatially correlated with a lower physiological expression of genes *DNASE1* and *CP*, two genes with potential implications for neurodegeneration. *DNASE1* encodes an enzyme responsible for DNA degradation and is involved in apoptosis and DNA clearance [89]. In the CNS, this gene is primarily expressed in neurons and astrocytes, where it is involved in apoptotic DNA fragmentation in mature neurons [90]. Lower expression of *DNASE1* in specific DMN regions may impair DNA clearance, driving chronic inflammation and affecting long-range connections.

Conversely, *CP* encodes ceruloplasmin, a protein involved in copper and iron homeostasis. In the CNS, it regulates iron metabolism [91]. Iron accumulation in MS contributes to neurotoxicity through oxidative stress and ferroptosis [92]. Higher levels of ceruloplasmin in astrocytes and axons have been found in periplaque WM, indicating that astrocytes may be responsible for the efflux and the accumulation of iron in areas of slowly expanding lesions, therefore promoting demyelination and neurodegeneration [93]. Regions with lower physiological *CP* expression may have a more limited iron metabolism, potentially reducing their susceptibility to iron-driven neurotoxicity.

Cognitively impaired MS patients also showed lower centrality in small clusters of the deep GM nuclei and SMN. This pattern was previously described [7, 94], but no significant cross-correlations with transcriptomic data were observed. This may be due to the smaller, more dispersed abnormality maps and the heterogeneous microarray sampling in the AHBA dataset among individual donors.

Of note, RRMS patients and cognitively preserved MS patients show no significant suprathreshold abnormalities consistent with early, subtle RSN reorganization [12, 95] that becomes more evident in advanced disease [7].

Interestingly, our results, when considered alongside a previous study that employed similar methods to investigate GM atrophy in MS [35], provide additional insights into the potential relationship between physiological gene expression and MRI abnormalities in MS. The previous study identified higher expression of genes related to GABA neurotransmission and lower expression of genes associated with mitochondrial function in regions exhibiting GM atrophy [35]. In contrast, our results demonstrated increased expression of mitochondria-related genes in regions with higher centrality in PMS patients. This suggests that brain regions naturally more vulnerable to mitochondrial dysfunction may be more prone to GM atrophy, whereas regions with greater energy production capacity may be relatively preserved, leading to higher centrality. The absence of other overlapping enriched pathways between the two studies highlights the complex and heterogeneous nature of MS pathology, suggesting that distinct structural and functional abnormalities may arise from different underlying biological mechanisms.

Our study has several limitations. First, we cannot establish a causal relationship between specific gene expression and RSN reorganization, since our MRI data were derived in vivo from MS patients and HC, whereas the AHBA dataset is based on post-mortem brains. Accordingly, our findings only suggest potential associations between regional gene expression and the susceptibility of different brain areas to MS-related pathological abnormalities. Second, regional gene expression may be influenced by MS pathology or DMTs, which could not be explored in our study because transcriptomic data were only available from HC. The potential impact of MS-related pathology or pharmacological interventions on gene expression needs to be explored in future studies. Third, cognitive data were available only for a subgroup of MS patients, reducing the sensitivity of our analysis. However, this subgroup represents approximately 88% of the MS study population, making it a representative sample. Fourth, MRI acquisitions were obtained from two different scanners. However, voxel-wise degree centrality analyses have been successfully conducted in multicentre studies without significant harmonization issues [25], and all our MRI analyses were statistically corrected for scanner-related differences. Fifth, the location and distribution of MS-related focal and diffuse WM and GM damage can strongly influence disease-specific patterns of functional reorganization, thus contributing to the functional network abnormalities observed in our study [96, 97]. Even a single strategically located WM lesion can trigger widespread connectivity alterations [96], whereas specific spatial patterns of WM lesions may be associated with distinct regional cortical atrophy, predicting different risk of disability progression [97], and possibly also promoting heterogeneous RSN reorganization. In contrast, our study is based on physiological spatial gene expression, which may identify brain regions that are intrinsically more or less vulnerable to functional alteration. Thus, while the spatial transcriptomic signatures we identify might represent a biological predisposition for network reorganization, the actual functional abnormalities observed in MS are likely shaped by the additional effects of lesions, microstructural abnormalities, and atrophy. Future studies should further explore how intrinsic biological factors interact with MS-related pathological processes to drive the observed fMRI–gene expression relationships.

Sixth, we chose a liberal threshold (p < 0.01, uncorrected) for initial spatial cross-correlations. However, this approach is commonly used in studies employing AHBA [37, 49, 50], as large regions of interest are necessary due to the limited sampling density of brain donors. Moreover, the risk of false positives was minimized through second-level analyses employing stringent statistical corrections. Finally, even though age was included as a covariate in all voxel-wise and conventional MRI analyses, residual confounding due to age cannot be completely excluded.

In conclusion, our findings support the hypothesis that RSN reorganization in MS is influenced by the intrinsic resilience of brain hubs, mediated by regional gene expression. Our work provides valuable insights into the molecular pathways contributing to functional network dysfunction in MS. Future research should aim to establish causal relationships and identify potential therapeutic targets to mitigate maladaptive network changes associated with clinical disability and cognitive impairment in MS.

## Supplementary information

Supplementary material

## Data Availability

Prof. Rocca has full access to all the data in the study and takes responsibility for the integrity of the data and the accuracy of the data analysis.

## References

[1] Kuhlmann T, Moccia M, Coetzee T, Cohen JA, Correale J, Graves J, et al. Multiple sclerosis progression: time for a new mechanism-driven framework. Lancet Neurol. 2023;22:78–88. 10.1016/S1474-4422(22)00289-7. 36410373 10.1016/S1474-4422(22)00289-7 PMC10463558

[2] Filippi M, Bar-Or A, Piehl F, Preziosa P, Solari A, Vukusic S, et al. Multiple sclerosis. Nat Rev Dis Primers. 2018;4:43 10.1038/s41572-018-0041-4. 30410033 10.1038/s41572-018-0041-4

[3] Filippi M, Bruck W, Chard D, Fazekas F, Geurts JJG, Enzinger C, et al. Association between pathological and MRI findings in multiple sclerosis. Lancet Neurol. 2019;18:198–210. 10.1016/S1474-4422(18)30451-4. 30663609 10.1016/S1474-4422(18)30451-4

[4] Preziosa P, Valsasina P, Filippi M, Rocca MA Human Functional MRI. In: Groppa S, G. Meuth S, editors. Translational Methods for Multiple Sclerosis Research. Neuromethods. New York, NY: Springer US; 2021. p. 213-36.

[5] Rocca MA, Schoonheim MM, Valsasina P, Geurts JJG, Filippi M. Task- and resting-state fMRI studies in multiple sclerosis: from regions to systems and time-varying analysis. Current status and future perspective. Neuroimage Clin. 2022;35:103076. 10.1016/j.nicl.2022.103076. 35691253 10.1016/j.nicl.2022.103076 PMC9194954

[6] Meijer KA, Eijlers AJC, Geurts JJG, Schoonheim MM. Staging of cortical and deep grey matter functional connectivity changes in multiple sclerosis. J Neurol Neurosurg Psychiatry. 2018;89:205–10. 10.1136/jnnp-2017-316329. 28986469 10.1136/jnnp-2017-316329

[7] Rocca MA, Valsasina P, Leavitt VM, Rodegher M, Radaelli M, Riccitelli GC, et al. Functional network connectivity abnormalities in multiple sclerosis: correlations with disability and cognitive impairment. Mult Scler. 2018;24:459–71. 10.1177/1352458517699875. 28294693 10.1177/1352458517699875

[8] Dogonowski AM, Siebner HR, Soelberg Sorensen P, Paulson OB, Dyrby TB, Blinkenberg M, et al. Resting-state connectivity of pre-motor cortex reflects disability in multiple sclerosis. Acta Neurol Scand. 2013;128:328–35. 10.1111/ane.12121. 23461607 10.1111/ane.12121

[9] Zhong J, Nantes JC, Holmes SA, Gallant S, Narayanan S, Koski L. Abnormal functional connectivity and cortical integrity influence dominant hand motor disability in multiple sclerosis: a multimodal analysis. Hum Brain Mapp. 2016;37:4262–75. 10.1002/hbm.23307. 27381089 10.1002/hbm.23307 PMC6867582

[10] Sbardella E, Upadhyay N, Tona F, Prosperini L, De Giglio L, Petsas N, et al. Dentate nucleus connectivity in adult patients with multiple sclerosis: functional changes at rest and correlation with clinical features. Mult Scler. 2017;23:546–55. 10.1177/1352458516657438. 27411700 10.1177/1352458516657438

[11] Strik M, Chard DT, Dekker I, Meijer KA, Eijlers AJ, Pardini M, et al. Increased functional sensorimotor network efficiency relates to disability in multiple sclerosis. Mult Scler. 2021;27:1364–73. 10.1177/1352458520966292. 33104448 10.1177/1352458520966292 PMC8358536

[12] Faivre A, Rico A, Zaaraoui W, Crespy L, Reuter F, Wybrecht D, et al. Assessing brain connectivity at rest is clinically relevant in early multiple sclerosis. Mult Scler. 2012;18:1251–8. 10.1177/1352458511435930. 22307385 10.1177/1352458511435930

[13] Tommasin S, De Giglio L, Ruggieri S, Petsas N, Gianni C, Pozzilli C, et al. Multi-scale resting state functional reorganization in response to multiple sclerosis damage. Neuroradiology. 2020;62:693–704. 10.1007/s00234-020-02393-0. 32189024 10.1007/s00234-020-02393-0

[14] Rocca MA, Valsasina P, Absinta M, Riccitelli G, Rodegher ME, Misci P, et al. Default-mode network dysfunction and cognitive impairment in progressive MS. Neurology. 2010;74:1252–9. 10.1212/WNL.0b013e3181d9ed91. 20404306 10.1212/WNL.0b013e3181d9ed91

[15] Meijer KA, Eijlers AJC, Douw L, Uitdehaag BMJ, Barkhof F, Geurts JJG, et al. Increased connectivity of hub networks and cognitive impairment in multiple sclerosis. Neurology. 2017;88:2107–14. 10.1212/WNL.0000000000003982. 28468841 10.1212/WNL.0000000000003982

[16] Jandric D, Lipp I, Paling D, Rog D, Castellazzi G, Haroon H, et al. Mechanisms of network changes in cognitive impairment in multiple sclerosis. Neurology. 2021;97:e1886–e97. 10.1212/WNL.0000000000012834. 34649879 10.1212/WNL.0000000000012834 PMC8601205

[17] Marchesi O, Bonacchi R, Valsasina P, Preziosa P, Pagani E, Cacciaguerra L, et al. Functional and structural MRI correlates of executive functions in multiple sclerosis. Mult Scler. 2022;28:742–56. 10.1177/13524585211033184. 34387534 10.1177/13524585211033184

[18] Karavasilis E, Christidi F, Velonakis G, Tzanetakos D, Zalonis I, Potagas C, et al. Hippocampal structural and functional integrity in multiple sclerosis patients with or without memory impairment: a multimodal neuroimaging study. Brain Imaging Behav. 2019;13:1049–59. 10.1007/s11682-018-9924-y. 29971687 10.1007/s11682-018-9924-y

[19] Rubinov M, Sporns O. Complex network measures of brain connectivity: uses and interpretations. Neuroimage. 2010;52:1059–69. 10.1016/j.neuroimage.2009.10.003. 19819337 10.1016/j.neuroimage.2009.10.003

[20] Shu N, Duan Y, Xia M, Schoonheim MM, Huang J, Ren Z, et al. Disrupted topological organization of structural and functional brain connectomes in clinically isolated syndrome and multiple sclerosis. Sci Rep. 2016;6:29383. 10.1038/srep29383. 27403924 10.1038/srep29383 PMC4941534

[21] Rocca MA, Valsasina P, Meani A, Falini A, Comi G, Filippi M. Impaired functional integration in multiple sclerosis: a graph theory study. Brain Struct Funct. 2016;221:115–31. 10.1007/s00429-014-0896-4. 25257603 10.1007/s00429-014-0896-4

[22] Liu Y, Wang H, Duan Y, Huang J, Ren Z, Ye J, et al. Functional brain network alterations in clinically isolated syndrome and multiple sclerosis: a graph-based connectome study. Radiology. 2017;282:534–41. 10.1148/radiol.2016152843. 27541686 10.1148/radiol.2016152843

[23] Hejazi S, Karwowski W, Farahani FV, Marek T, Hancock PA. Graph-Based analysis of brain connectivity in multiple sclerosis using functional MRI: a systematic review. Brain Sci. 2023;13:246. 10.3390/brainsci13020246. 36831789 10.3390/brainsci13020246 PMC9953947

[24] Gamboa OL, Tagliazucchi E, von Wegner F, Jurcoane A, Wahl M, Laufs H, et al. Working memory performance of early MS patients correlates inversely with modularity increases in resting state functional connectivity networks. Neuroimage. 2014;94:385–95. 10.1016/j.neuroimage.2013.12.008. 24361662 10.1016/j.neuroimage.2013.12.008

[25] Carotenuto A, Valsasina P, Schoonheim MM, Geurts JJG, Barkhof F, Gallo A, et al. Investigating functional network abnormalities and associations with disability in multiple sclerosis. Neurology. 2022;99:e2517–e30. 10.1212/WNL.0000000000201264. 36096690 10.1212/WNL.0000000000201264

[26] Zuo XN, Ehmke R, Mennes M, Imperati D, Castellanos FX, Sporns O, et al. Network centrality in the human functional connectome. Cereb Cortex. 2012;22:1862–75. 10.1093/cercor/bhr269. 21968567 10.1093/cercor/bhr269

[27] Schoonheim MM, Geurts J, Wiebenga OT, De Munck JC, Polman CH, Stam CJ, et al. Changes in functional network centrality underlie cognitive dysfunction and physical disability in multiple sclerosis. Mult Scler. 2014;20:1058–65. 10.1177/1352458513516892. 24347184 10.1177/1352458513516892

[28] Eijlers AJ, Meijer KA, Wassenaar TM, Steenwijk MD, Uitdehaag BM, Barkhof F, et al. Increased default-mode network centrality in cognitively impaired multiple sclerosis patients. Neurology. 2017;88:952–60. 10.1212/WNL.0000000000003689. 28179464 10.1212/WNL.0000000000003689

[29] Dekker I, Schoonheim MM, Venkatraghavan V, Eijlers AJC, Brouwer I, Bron EE, et al. The sequence of structural, functional and cognitive changes in multiple sclerosis. Neuroimage Clin. 2021;29:102550. 10.1016/j.nicl.2020.102550. 33418173 10.1016/j.nicl.2020.102550 PMC7804841

[30] Hawellek DJ, Hipp JF, Lewis CM, Corbetta M, Engel AK. Increased functional connectivity indicates the severity of cognitive impairment in multiple sclerosis. Proc Natl Acad Sci USA. 2011;108:19066–71. 10.1073/pnas.1110024108. 22065778 10.1073/pnas.1110024108 PMC3223469

[31] Schoonheim MM, Meijer KA, Geurts JJ. Network collapse and cognitive impairment in multiple sclerosis. Front Neurol. 2015;6:82 10.3389/fneur.2015.00082. 25926813 10.3389/fneur.2015.00082 PMC4396388

[32] Hawrylycz MJ, Lein ES, Guillozet-Bongaarts AL, Shen EH, Ng L, Miller JA, et al. An anatomically comprehensive atlas of the adult human brain transcriptome. Nature. 2012;489:391–9. 10.1038/nature11405. 22996553 10.1038/nature11405 PMC4243026

[33] Arnatkeviciute A, Fulcher BD, Bellgrove MA, Fornito A. Imaging transcriptomics of brain disorders. Biol Psychiatry Glob Open Sci. 2022;2:319–31. 10.1016/j.bpsgos.2021.10.002. 36324650 10.1016/j.bpsgos.2021.10.002 PMC9616271

[34] Diez I, Sepulcre J. Unveiling the neuroimaging-genetic intersections in the human brain. Curr Opin Neurol. 2021;34:480–7. 10.1097/WCO.0000000000000952. 34227572 10.1097/WCO.0000000000000952 PMC8265485

[35] Preziosa P, Storelli L, Tedone N, Margoni M, Mistri D, Azzimonti M, et al. Spatial correspondence among regional gene expressions and gray matter volume loss in multiple sclerosis. Mol Psychiatry. 2024;29:1833–43. 10.1038/s41380-024-02452-5. 38326561 10.1038/s41380-024-02452-5

[36] Cacciaguerra L, Storelli L, Pagani E, Preziosa P, Mesaros S, Martinelli V, et al. Use of brain MRI and gene expression atlases to reconstruct the pathophysiology of autoimmune neurological disorders: The proof-of-concept of NMOSD. Mult Scler. 2025;31:140–58. 10.1177/13524585241307154. 39891565 10.1177/13524585241307154 PMC11789429

[37] Sun J, Guo M, Chai L, Xu S, Lizhu Y, Li Y, et al. Distinct virtual histology of grey matter atrophy in four neuroinflammatory diseases. Brain. 2024;147:3906–17. 10.1093/brain/awae138. 38703370 10.1093/brain/awae138

[38] Oldfield RC. The assessment and analysis of handedness: the Edinburgh inventory. Neuropsychologia. 1971;9:97–113. 10.1016/0028-3932(71)90067-4. 5146491 10.1016/0028-3932(71)90067-4

[39] Thompson AJ, Banwell BL, Barkhof F, Carroll WM, Coetzee T, Comi G, et al. Diagnosis of multiple sclerosis: 2017 revisions of the McDonald criteria. Lancet Neurol. 2018;17:162–73. 10.1016/S1474-4422(17)30470-2. 29275977 10.1016/S1474-4422(17)30470-2

[40] Kurtzke JF. Rating neurologic impairment in multiple sclerosis: an expanded disability status scale (EDSS). Neurology. 1983;33:1444–52. 10.1212/wnl.33.11.1444. 6685237 10.1212/wnl.33.11.1444

[41] Rao SM, Leo GJ, Haughton VM, St Aubin-Faubert P, Bernardin L. Correlation of magnetic resonance imaging with neuropsychological testing in multiple sclerosis. Neurology. 1989;39:161–6. 10.1212/wnl.39.2.161. 2915783 10.1212/wnl.39.2.161

[42] Tedone N, Vizzino C, Meani A, Gallo A, Altieri M, D’Ambrosio A, et al. The brief repeatable battery of neuropsychological tests (BRB-N) version a: update of Italian normative data from the Italian neuroimaging network initiative (INNI). J Neurol. 2024;271:1813–23. 10.1007/s00415-023-12108-z. 38060030 10.1007/s00415-023-12108-z

[43] Amato MP, Morra VB, Falautano M, Ghezzi A, Goretti B, Patti F, et al. Cognitive assessment in multiple sclerosis-an Italian consensus. Neurol Sci. 2018;39:1317–24. 10.1007/s10072-018-3427-x. 29766386 10.1007/s10072-018-3427-x

[44] Valverde S, Cabezas M, Roura E, Gonzalez-Villa S, Pareto D, Vilanova JC, et al. Improving automated multiple sclerosis lesion segmentation with a cascaded 3D convolutional neural network approach. Neuroimage. 2017;155:159–68. 10.1016/j.neuroimage.2017.04.034. 28435096 10.1016/j.neuroimage.2017.04.034

[45] Battaglini M, Jenkinson M, De Stefano N. Alzheimer’s Disease Neuroimaging I. SIENA-XL for improving the assessment of gray and white matter volume changes on brain MRI. Hum Brain Mapp. 2018;39:1063–77. 10.1002/hbm.23828. 29222814 10.1002/hbm.23828 PMC6866496

[46] Zhuang Y, Zhou F, Gong H. Intrinsic functional plasticity of the sensorimotor network in relapsing-remitting multiple sclerosis: evidence from a centrality analysis. PLoS One. 2015;10:e0130524. 10.1371/journal.pone.0130524. 26110420 10.1371/journal.pone.0130524 PMC4482320

[47] Friston KJ, Holmes AP, Price CJ, Buchel C, Worsley KJ. Multisubject fMRI studies and conjunction analyses. Neuroimage. 1999;10:385–96. 10.1006/nimg.1999.0484. 10493897 10.1006/nimg.1999.0484

[48] Holmes AP, Friston KJ. Generalisability, Random Effects & Population Inference. NeuroImage. 1998;7:S754–S. 10.1016/s1053-8119(18)31587-8.

[49] Freeze B, Acosta D, Pandya S, Zhao Y, Raj A. Regional expression of genes mediating trans-synaptic alpha-synuclein transfer predicts regional atrophy in Parkinson disease. Neuroimage Clin. 2018;18:456–66. 10.1016/j.nicl.2018.01.009. 29868450 10.1016/j.nicl.2018.01.009 PMC5984599

[50] Writing Committee for the Attention-Deficit/Hyperactivity Disorder; Autism Spectrum Disorder; Bipolar Disorder; Major Depressive Disorder; Obsessive-Compulsive Disorder; and Schizophrenia ENIGMA Working Groups, Patel Y, Parker N, Shin J, Howard D. Virtual Histology of Cortical Thickness and Shared Neurobiology in 6 Psychiatric Disorders. JAMA Psychiatry. 2021;78:47–63. 10.1001/jamapsychiatry.2020.2694. 32857118 10.1001/jamapsychiatry.2020.2694 PMC7450410

[51] Rizzo G, Veronese M, Expert P, Turkheimer FE, Bertoldo A. MENGA: a new comprehensive tool for the integration of neuroimaging data and the allen human brain transcriptome atlas. PLoS One. 2016;11:e0148744. 10.1371/journal.pone.0148744. 26882227 10.1371/journal.pone.0148744 PMC4755531

[52] Arnatkeviciute A, Fulcher BD, Fornito A. A practical guide to linking brain-wide gene expression and neuroimaging data. Neuroimage. 2019;189:353–67. 10.1016/j.neuroimage.2019.01.011. 30648605 10.1016/j.neuroimage.2019.01.011

[53] Chen J, Bardes EE, Aronow BJ, Jegga AG. ToppGene Suite for gene list enrichment analysis and candidate gene prioritization. Nucleic Acids Res. 2009;37:W305–11. 10.1093/nar/gkp427. 19465376 10.1093/nar/gkp427 PMC2703978

[54] McKenzie AT, Wang M, Hauberg ME, Fullard JF, Kozlenkov A, Keenan A, et al. Brain cell type specific gene expression and co-expression network architectures. Sci Rep. 2018;8:8868. 10.1038/s41598-018-27293-5. 29892006 10.1038/s41598-018-27293-5 PMC5995803

[55] Darmanis S, Sloan SA, Zhang Y, Enge M, Caneda C, Shuer LM, et al. A survey of human brain transcriptome diversity at the single cell level. Proc Natl Acad Sci USA. 2015;112:7285–90. 10.1073/pnas.1507125112. 26060301 10.1073/pnas.1507125112 PMC4466750

[56] Zhao Y, Wong L, Goh WWB. How to do quantile normalization correctly for gene expression data analyses. Sci Rep. 2020;10:15534. 10.1038/s41598-020-72664-6. 32968196 10.1038/s41598-020-72664-6 PMC7511327

[57] De Stefano N, Giorgio A, Battaglini M, Rovaris M, Sormani MP, Barkhof F, et al. Assessing brain atrophy rates in a large population of untreated multiple sclerosis subtypes. Neurology. 2010;74:1868–76. 10.1212/WNL.0b013e3181e24136. 20530323 10.1212/WNL.0b013e3181e24136

[58] Fisher E, Lee JC, Nakamura K, Rudick RA. Gray matter atrophy in multiple sclerosis: a longitudinal study. Ann Neurol. 2008;64:255–65. 10.1002/ana.21436. 18661561 10.1002/ana.21436

[59] Rocca MA, Valsasina P, Meani A, Gobbi C, Zecca C, Rovira A, et al. Association of gray matter atrophy patterns with clinical phenotype and progression in multiple sclerosis. Neurology. 2021;96:e1561–e73. 10.1212/WNL.0000000000011494. 33441452 10.1212/WNL.0000000000011494

[60] Arnett PA, Rao SM, Bernardin L, Grafman J, Yetkin FZ, Lobeck L. Relationship between frontal lobe lesions and wisconsin card sorting test performance in patients with multiple sclerosis. Neurology. 1994;44:420–5. 10.1212/wnl.44.3_part_1.420. 8145908 10.1212/wnl.44.3_part_1.420

[61] Sepulcre J, Masdeu JC, Pastor MA, Goni J, Barbosa C, Bejarano B, et al. Brain pathways of verbal working memory: a lesion-function correlation study. Neuroimage. 2009;47:773–8. 10.1016/j.neuroimage.2009.04.054. 19393745 10.1016/j.neuroimage.2009.04.054

[62] Kincses ZT, Ropele S, Jenkinson M, Khalil M, Petrovic K, Loitfelder M, et al. Lesion probability mapping to explain clinical deficits and cognitive performance in multiple sclerosis. Mult Scler. 2011;17:681–9. 10.1177/1352458510391342. 21177325 10.1177/1352458510391342

[63] Filippi M, Preziosa P, Copetti M, Riccitelli G, Horsfield MA, Martinelli V, et al. Gray matter damage predicts the accumulation of disability 13 years later in MS. Neurology. 2013;81:1759–67. 10.1212/01.wnl.0000435551.90824.d0. 24122185 10.1212/01.wnl.0000435551.90824.d0

[64] Huiskamp M, Eijlers AJC, Broeders TAA, Pasteuning J, Dekker I, Uitdehaag BMJ, et al. Longitudinal network changes and conversion to cognitive impairment in multiple sclerosis. Neurology. 2021;97:e794–e802. 10.1212/WNL.0000000000012341. 34099528 10.1212/WNL.0000000000012341 PMC8397585

[65] Tejera-Alhambra M, Casrouge A, de Andres C, Ramos-Medina R, Alonso B, Vega J, et al. Low DPP4 expression and activity in multiple sclerosis. Clin Immunol. 2014;150:170–83. 10.1016/j.clim.2013.11.011. 24412911 10.1016/j.clim.2013.11.011

[66] Kushner SA, Elgersma Y, Murphy GG, Jaarsma D, van Woerden GM, Hojjati MR, et al. Modulation of presynaptic plasticity and learning by the H-ras/extracellular signal-regulated kinase/synapsin I signaling pathway. J Neurosci. 2005;25:9721–34. 10.1523/JNEUROSCI.2836-05.2005. 16237176 10.1523/JNEUROSCI.2836-05.2005 PMC2802213

[67] Doroszkiewicz J, Kulczynska-Przybik A, Dulewicz M, Borawska R, Zajkowska M, Slowik A, et al. Potential utility of cerebrospinal fluid glycoprotein nonmetastatic melanoma protein b as a neuroinflammatory diagnostic biomarker in mild cognitive impairment and alzheimer’s disease. J Clin Med. 2023;12:4689 10.3390/jcm12144689. 37510803 10.3390/jcm12144689 PMC10380476

[68] Hendrickx DAE, van Scheppingen J, van der Poel M, Bossers K, Schuurman KG, van Eden CG, et al. Gene expression profiling of multiple sclerosis pathology identifies early patterns of demyelination surrounding chronic active lesions. Front Immunol. 2017;8:1810 10.3389/fimmu.2017.01810. 29312322 10.3389/fimmu.2017.01810 PMC5742619

[69] Chesik D, Wilczak N, De Keyser J. The insulin-like growth factor system in multiple sclerosis. Int Rev Neurobiol. 2007;79:203–26. 10.1016/S0074-7742(07)79009-8. 17531843 10.1016/S0074-7742(07)79009-8

[70] Evans R, Watkins LM, Hawkins K, Santiago G, Demetriou C, Naughton M, et al. Complement activation and increased anaphylatoxin receptor expression are associated with cortical grey matter lesions and the compartmentalised inflammatory response of multiple sclerosis. Front Cell Neurosci. 2023;17:1094106. 10.3389/fncel.2023.1094106. 37032838 10.3389/fncel.2023.1094106 PMC10073739

[71] Conti P, Kempuraj D. Important role of mast cells in multiple sclerosis. Mult Scler Relat Disord. 2016;5:77–80. 10.1016/j.msard.2015.11.005. 26856948 10.1016/j.msard.2015.11.005

[72] Kaplan L, Chow BW, Gu C. Neuronal regulation of the blood-brain barrier and neurovascular coupling. Nat Rev Neurosci. 2020;21:416–32. 10.1038/s41583-020-0322-2. 32636528 10.1038/s41583-020-0322-2 PMC8934575

[73] Cashion JM, Young KM, Sutherland BA. How does neurovascular unit dysfunction contribute to multiple sclerosis?. Neurobiol Dis. 2023;178:106028. 10.1016/j.nbd.2023.106028. 36736923 10.1016/j.nbd.2023.106028

[74] Buxton RB, Uludag K, Dubowitz DJ, Liu TT. Modeling the hemodynamic response to brain activation. Neuroimage. 2004;23:S220–33. 10.1016/j.neuroimage.2004.07.013. 15501093 10.1016/j.neuroimage.2004.07.013

[75] Dogonowski AM, Andersen KW, Madsen KH, Sorensen PS, Paulson OB, Blinkenberg M, et al. Multiple sclerosis impairs regional functional connectivity in the cerebellum. Neuroimage Clin. 2014;4:130–8. 10.1016/j.nicl.2013.11.005. 24371795 10.1016/j.nicl.2013.11.005 PMC3871286

[76] Woo MS, Engler JB, Friese MA. The neuropathobiology of multiple sclerosis. Nat Rev Neurosci. 2024;25:493–513. 10.1038/s41583-024-00823-z. 38789516 10.1038/s41583-024-00823-z

[77] Becher B, Spath S, Goverman J. Cytokine networks in neuroinflammation. Nat Rev Immunol. 2017;17:49–59. 10.1038/nri.2016.123. 27916979 10.1038/nri.2016.123

[78] Haider L, Zrzavy T, Hametner S, Hoftberger R, Bagnato F, Grabner G, et al. The topograpy of demyelination and neurodegeneration in the multiple sclerosis brain. Brain. 2016;139:807–15. 10.1093/brain/awv398. 26912645 10.1093/brain/awv398 PMC4766379

[79] Howell OW, Schulz-Trieglaff EK, Carassiti D, Gentleman SM, Nicholas R, Roncaroli F, et al. Extensive grey matter pathology in the cerebellum in multiple sclerosis is linked to inflammation in the subarachnoid space. Neuropathol Appl Neurobiol. 2015;41:798–813. 10.1111/nan.12199. 25421634 10.1111/nan.12199

[80] Basile B, Castelli M, Monteleone F, Nocentini U, Caltagirone C, Centonze D, et al. Functional connectivity changes within specific networks parallel the clinical evolution of multiple sclerosis. Mult Scler. 2014;20:1050–7. 10.1177/1352458513515082. 24326671 10.1177/1352458513515082

[81] Mehler MF. Epigenetic principles and mechanisms underlying nervous system functions in health and disease. Prog Neurobiol. 2008;86:305–41. 10.1016/j.pneurobio.2008.10.001. 18940229 10.1016/j.pneurobio.2008.10.001 PMC2636693

[82] Gapp K, Woldemichael BT, Bohacek J, Mansuy IM. Epigenetic regulation in neurodevelopment and neurodegenerative diseases. Neuroscience. 2014;264:99–111. 10.1016/j.neuroscience.2012.11.040. 23256926 10.1016/j.neuroscience.2012.11.040

[83] Telese F, Gamliel A, Skowronska-Krawczyk D, Garcia-Bassets I, Rosenfeld MG. Seq-ing” insights into the epigenetics of neuronal gene regulation. Neuron. 2013;77:606–23. 10.1016/j.neuron.2013.01.034. 23439116 10.1016/j.neuron.2013.01.034 PMC3736682

[84] Palombit A, Silvestri E, Volpi T, Aiello M, Cecchin D, Bertoldo A, et al. Variability of regional glucose metabolism and the topology of functional networks in the human brain. Neuroimage. 2022;257:119280. 10.1016/j.neuroimage.2022.119280. 35525522 10.1016/j.neuroimage.2022.119280

[85] Eisenberg E, Levanon EY. Human housekeeping genes, revisited. Trends Genet. 2013;29:569–74. 10.1016/j.tig.2013.05.010. 23810203 10.1016/j.tig.2013.05.010

[86] Filippi M, Preziosa P, Barkhof F, Ciccarelli O, Cossarizza A, De Stefano N, et al. The ageing central nervous system in multiple sclerosis: the imaging perspective. Brain. 2024;147:3665–80. 10.1093/brain/awae251. 39045667 10.1093/brain/awae251 PMC11531849

[87] Hulst HE, Schoonheim MM, Van Geest Q, Uitdehaag BM, Barkhof F, Geurts JJ. Memory impairment in multiple sclerosis: Relevance of hippocampal activation and hippocampal connectivity. Mult Scler. 2015;21:1705–12. 10.1177/1352458514567727. 25680986 10.1177/1352458514567727

[88] Rocca MA, Valsasina P, Hulst HE, Abdel-Aziz K, Enzinger C, Gallo A, et al. Functional correlates of cognitive dysfunction in multiple sclerosis: A multicenter fMRI Study. Hum Brain Mapp. 2014;35:5799–814. 10.1002/hbm.22586. 25045065 10.1002/hbm.22586 PMC6869325

[89] Keyel PA. Dnases in health and disease. Dev Biol. 2017;429:1–11. 10.1016/j.ydbio.2017.06.028. 28666955 10.1016/j.ydbio.2017.06.028 PMC11492367

[90] Shiokawa D, Tanuma S. Differential DNases are selectively used in neuronal apoptosis depending on the differentiation state. Cell Death Differ. 2004;11:1112–20. 10.1038/sj.cdd.4401454. 15167901 10.1038/sj.cdd.4401454

[91] Cheli VT, Sekhar M, Santiago Gonzalez DA, Angeliu CG, Denaroso GE, Smith Z, et al. The expression of ceruloplasmin in astrocytes is essential for postnatal myelination and myelin maintenance in the adult brain. Glia. 2023;71:2323–42. 10.1002/glia.24424. 37269227 10.1002/glia.24424 PMC10599212

[92] Zierfuss B, Wang Z, Jackson AN, Moezzi D, Yong VW. Iron in multiple sclerosis - Neuropathology, immunology, and real-world considerations. Mult Scler Relat Disord. 2023;78:104934. 10.1016/j.msard.2023.104934. 37579645 10.1016/j.msard.2023.104934

[93] Hametner S, Wimmer I, Haider L, Pfeifenbring S, Bruck W, Lassmann H. Iron and neurodegeneration in the multiple sclerosis brain. Ann Neurol. 2013;74:848–61. 10.1002/ana.23974. 23868451 10.1002/ana.23974 PMC4223935

[94] Tona F, Petsas N, Sbardella E, Prosperini L, Carmellini M, Pozzilli C, et al. Multiple sclerosis: altered thalamic resting-state functional connectivity and its effect on cognitive function. Radiology. 2014;271:814–21. 10.1148/radiol.14131688. 24484065 10.1148/radiol.14131688

[95] Roosendaal SD, Schoonheim MM, Hulst HE, Sanz-Arigita EJ, Smith SM, Geurts JJ, et al. Resting state networks change in clinically isolated syndrome. Brain. 2010;133:1612–21. 10.1093/brain/awq058. 20356855 10.1093/brain/awq058

[96] Droby A, Yuen KS, Muthuraman M, Reitz SC, Fleischer V, Klein J, et al. Changes in brain functional connectivity patterns are driven by an individual lesion in MS: a resting-state fMRI study. Brain Imaging Behav. 2016;10:1117–26. 10.1007/s11682-015-9476-3. 26553579 10.1007/s11682-015-9476-3

[97] Muthuraman M, Fleischer V, Kroth J, Ciolac D, Radetz A, Koirala N, et al. Covarying patterns of white matter lesions and cortical atrophy predict progression in early MS. Neurol Neuroimmunol Neuroinflamm. 2020;7:e681. 10.1212/NXI.0000000000000681. 32024782 10.1212/NXI.0000000000000681 PMC7051213

