# Supplementary material for: Spatial gene expression and functional network abnormalities in multiple sclerosis: exploring biological influence on brain functional reorganization
Source: Transl Psychiatry. 2026 Mar 3;16:137. doi: 10.1038/s41398-026-03921-x (PMC12982597; doi:10.1038/s41398-026-03921-x)
Supplement: Supplementary file 1 — Supplementary material [file 41398_2026_3921_MOESM1_ESM.docx]

**Supplementary material for the manuscript entitled:**

Spatial gene expression and functional network abnormalities in multiple sclerosis: exploring biological influence on brain functional reorganization

Authors: Paolo Preziosa, Matteo Azzimonti, Loredana Storelli, Paola Valsasina, Nicolò Tedone, Monica Margoni, Massimo Filippi, Maria A. Rocca

This file contains Supplementary methods, 5 Supplementary Tables.

File format: .docx

**Supplementary Methods**

MRI acquisition protocol. Images acquired using Scanner 1 included the following protocol: 1) 200 sets of T2*-weighted echo planar imaging (EPI) sequence for resting state (RS) functional MRI (fMRI) (repetition time [TR]=3000 milliseconds; echo time [TE]=35 milliseconds, flip angle=90°, matrix size=128x128; field of view [FOV]=240 mm^2^; 30 contiguous axial slices, 4 mm thick); 2) dual-echo turbo spin echo (TR=2599; TE=16-80 milliseconds; flip angle=90°; matrix size=256×256; FOV=240 mm^2^; echo train length [ETL]=6; 44 contiguous axial slices, 3 mm thick); 3) Three-dimensional (3D) T1-weighted fast field echo (TR=25; TE=4.6 milliseconds; flip angle=30°; matrix size=256x256; FOV=230 mm^2^; 220 contiguous axial slices, 0.8 mm thick). Images acquired with Scanner 2 included: 1) 320 sets of T2*-weighted EPI sequence for RS fMRI (TR=1567 milliseconds; TE=35 milliseconds, flip angle=70°; multi-band factor=2, matrix size=96×96; FOV=240 x 240 mm^2^; 48 contiguous axial slices, 3 mm thick); 2) variable flip angle 3D T2-weighted fluid attenuated inversion recovery (FLAIR) (TR=4800 milliseconds; TE=270 milliseconds; inversion time [TI]=1650 milliseconds; matrix size=256 × 256; FOV=256 × 256 mm^2^; ETL=167; 192 contiguous sagittal slices, 1 mm thick); 3) variable flip angle 3D T2-weighted turbo spin echo (TR=2500 milliseconds; TE=330 milliseconds; matrix size=256 × 256; FOV=256 × 256 mm^2^; ETL = 117; 192 contiguous sagittal slices, 1 mm thick); 4) 3D T1-weighted turbo field echo (TR=7 milliseconds; TE=3.2 milliseconds; TI=1000 milliseconds; flip angle=8°; matrix size=256 × 256; FOV=256 × 256 mm^2^; 204 contiguous sagittal slices, 1 mm thick).

RS fMRI preprocessing. RS fMRI data processing was performed using the CONN toolbox [1]. RS fMRI images were realigned to the mean of each session using a rigid-body transformation to correct for head movements. After rigid registration of realigned images to the lesion-filled 3D T1-weighted scans, RS fMRI images were normalized to the Montreal Neurological Institute (MNI) space using a nonlinear transformation. After visual check of registration outputs and detection of outliers using the ART tool, the images were smoothed with a 6-mm^3^ Gaussian filter. For denoising, the first five principal components from CSF and WM were used as nuisance covariates, in accordance with the anatomical component-based noise correction method (aCompCor) [2]. The six rigid motion parameters and their first temporal derivatives were regressed out from data. Outliers detected by the ART toolbox (if any), along with spurious effects from the first two time points (to maximize magnetic equilibrium), were also regressed out from data. Finally, RS fMRI time series were linearly detrended and bandpass filtered (0.01–0.1 Hz).

**Supplementary references**

1. Whitfield-Gabrieli S, Nieto-Castanon A. Conn: a functional connectivity toolbox for correlated and anticorrelated brain networks. Brain Connect. 2012;2(3):125-41. doi:10.1089/brain.2012.0073.

2. Behzadi Y, Restom K, Liau J, Liu TT. A component based noise correction method (CompCor) for BOLD and perfusion based fMRI. Neuroimage. 2007;37(1):90-101. doi:10.1016/j.neuroimage.2007.04.042.

3. Amato MP, Morra VB, Falautano M, Ghezzi A, Goretti B, Patti F, et al. Cognitive assessment in multiple sclerosis-an Italian consensus. Neurol Sci. 2018;39(8):1317-24. doi:10.1007/s10072-018-3427-x.

4. Tedone N, Vizzino C, Meani A, Gallo A, Altieri M, D'Ambrosio A, et al. The brief repeatable battery of neuropsychological tests (BRB-N) version a: update of Italian normative data from the Italian Neuroimaging Network Initiative (INNI). J Neurol. 2024;271(4):1813-23. doi:10.1007/s00415-023-12108-z.

**Supplementary Table 1: Neuropsychological findings of MS patients.**

| **Cognitive**  **domains** | **MS patients**  **(N=491)** | | **Cognitively preserved MS**  **(N=347)** | | **Cognitively impaired MS**  **(N=144)** | |
| --- | --- | --- | --- | --- | --- | --- |
|  | **N of impaired^•^**  **(%)** | **Z-score^#^**  **(SD)** | **N of impaired^•^**  **(%)** | **Z-score^#^**  **(SD)** | **N of impaired^•^**  **(%)** | **Z-score^#^**  **(SD)** |
| Verbal memory | 160  (33%) | -0.57  (1.11) | 42  (12%) | -0.12  (0.88) | 118  (82%) | -1.65  (0.85) |
| Visual memory | 83  (17%) | -0.34  (0.91) | 12  (3.5%) | 0.02  (0.73) | 71  (51%) | -1.22  (0.69) |
| Information processing speed/attention | 199  (43%) | -0.52  (0.99) | 79  (24%) | -0.19  (0.83) | 120  (88%) | -1.51  (0.75) |
| Verbal fluency | 78  (16%) | -0.45  (1.04) | 15  (4%) | -0.11  (0.90) | 63  (44%) | -1.24  (0.88) |
| Global cognition | 144  (29%) | -0.43  (0.77) | - | -0.10  (0.55) | - | -1.40  (0.49) |

^*^number of MS patients (frequency) with ≥1 abnormal neuropsychological tests of BRB-N for each cognitive domain [3].

^#^Mean (standard deviation) of *z*-scores according to the normative data of an Italian representative sample; global z-scores were obtained by averaging z-scores of cognitive domains [4].

Abbreviations: SD=standard deviation**.**

**Supplementary Table 2: Genes showing a significant and reliable spatial association (chance likelihood <0.001 and auto-correlation ≥0.2) and an adjusted cross-correlation with a R^2^ ≥0.15 with the t value map of higher centrality in MS patients compared to HC. Direction (slope) of the cross-correlations and frequencies of gene inclusion in ToppGene Suite Gene Ontology are also shown.**

| **Gene ID** | **Gene name** | **Gene**  **auto-correlation** | **Cross-correlation** | | | **ToppGene Suite GO** | | |
| --- | --- | --- | --- | --- | --- | --- | --- | --- |
|  |  |  | **R^2^-adjusted** | **Direction**  **(slope)** | **p value** | **Molecular function** | **Biological**  **process** | **Cellular component** |
| *DPP4* | Dipeptidyl peptidase 4 | 0.65 | 0.43 | 1 | <0.001 | 0 | 1 | 0 |
| *MCM10* | Minichromosome maintenance 10 replication initiation factor | 0.38 | 0.41 | 1 | <0.001 | 0 | 0 | 0 |
| *C3AR1* | Complement C3a receptor 1 | 0.34 | 0.39 | -1 | <0.001 | 0 | 1 | 0 |
| *NPS* | Neuropeptide S | 0.21 | 0.36 | -1 | <0.001 | 0 | 0 | 0 |
| *ISCA1* | Iron-sulfur cluster assembly 1 | 0.46 | 0.31 | -1 | <0.001 | 0 | 0 | 0 |
| *ZRANB1* | Zinc finger RANBP2-type containing 1 | 0.24 | 0.30 | -1 | <0.001 | 0 | 0 | 0 |
| *PPARD* | Peroxisome proliferator activated receptor delta | 0.35 | 0.28 | -1 | <0.001 | 0 | 1 | 0 |
| *GALC* | Galactosylceramidase | 0.39 | 0.25 | -1 | <0.001 | 0 | 0 | 0 |
| *IGFBP3* | Insulin like growth factor binding protein 3 | 0.20 | 0.23 | 1 | <0.001 | 0 | 1 | 0 |
| *CAMKK2* | Calcium/calmodulin dependent protein kinase kinase 2 | 0.35 | 0.23 | -1 | <0.001 | 1 | 0 | 0 |
| *PHKG2* | Phosphorylase kinase catalytic subunit gamma 2 | 0.32 | 0.22 | 1 | <0.001 | 1 | 0 | 0 |
| *KIT* | KIT proto-oncogene, receptor tyrosine kinase | 0.80 | 0.22 | -1 | <0.001 | 0 | 2 | 0 |
| *GPNMB* | Glycoprotein NMB | 0.35 | 0.20 | 1 | <0.001 | 0 | 1 | 0 |
| *HECTD3* | HECT domain E3 ubiquitin protein ligase 3 | 0.29 | 0.20 | -1 | <0.001 | 0 | 0 | 0 |
| *TAL1* | TAL bHLH transcription factor 1, erythroid differentiation factor | 0.55 | 0.19 | -1 | <0.001 | 0 | 1 | 0 |
| *MAOA* | Monoamine oxidase A | 0.69 | 0.18 | 1 | <0.001 | 0 | 0 | 0 |
| *HRAS* | HRas proto-oncogene, GTPase | 0.21 | 0.17 | 1 | <0.001 | 0 | 1 | 0 |

Abbreviations: HC=healthy controls; GO=gene ontology; MS=multiple sclerosis.

**Supplementary Table 3: Genes showing a significant and reliable spatial association (chance likelihood <0.001 and auto-correlation ≥0.2) and an adjusted cross-correlation with a R^2^ ≥0.15 with the t value map of lower centrality in MS patients compared to HC. Direction (slope) of the cross-correlations and frequencies of gene inclusion in ToppGene Suite Gene Ontology are also shown.**

| **Gene ID** | **Gene name** | **Gene**  **auto-correlation** | **Cross-correlation** | | | **ToppGene Suite GO** | | |
| --- | --- | --- | --- | --- | --- | --- | --- | --- |
|  |  |  | **R^2^-adjusted** | **Direction**  **(slope)** | **p value** | **Molecular function** | **Biological**  **process** | **Cellular component** |
| *TMEM232* | Transmembrane protein 232 | 0.42 | 0.33 | -1 | <0.001 | 0 | 0 | 0 |
| *CHI3L1* | Chitinase 3 like 1 | 0.71 | 0.22 | -1 | <0.001 | 1 | 0 | 0 |
| *COQ10B* | Coenzyme Q10B | 0.34 | 0.20 | -1 | <0.001 | 0 | 0 | 0 |
| *AXL* | AXL receptor tyrosine kinase | 0.20 | 0.17 | 1 | <0.001 | 1 | 0 | 0 |
| *GRB7* | Growth factor receptor bound protein 7 | 0.39 | 0.17 | 1 | <0.001 | 0 | 0 | 0 |
| *ZNF579* | Zinc finger protein 579 | 0.32 | 0.16 | -1 | <0.001 | 0 | 0 | 0 |
| *TNFRSF25* | TNF receptor superfamily member 25 | 0.63 | 0.16 | 1 | <0.001 | 1 | 0 | 0 |
| *CD226* | CD226 molecule | 0.43 | 0.15 | -1 | <0.001 | 0 | 0 | 0 |
| *FZD4* | Frizzled class receptor 4 | 0.41 | 0.15 | 1 | <0.001 | 1 | 0 | 0 |
| *MEFV* | MEFV innate immunity regulator, pyrin | 0.62 | 0.15 | -1 | <0.001 | 1 | 0 | 0 |

Abbreviations: HC=healthy controls; GO=gene ontology; MS=multiple sclerosis.

**Supplementary Table 4: Genes showing a significant and reliable spatial association (chance likelihood <0.001 and auto-correlation ≥0.2) and an adjusted cross-correlation with a R^2^ ≥0.15 with the t value map of higher centrality in PMS patients compared to RRMS and HC. Direction (slope) of the cross-correlations and frequencies of gene inclusion in ToppGene Suite Gene Ontology are also shown.**

| **Gene ID** | **Gene name** | **Gene**  **auto-correlation** | **Cross-correlation** | | | **ToppGene Suite GO** | | |
| --- | --- | --- | --- | --- | --- | --- | --- | --- |
|  |  |  | **R^2^-adjusted** | **Direction**  **(slope)** | **p value** | **Molecular function** | **Biological**  **process** | **Cellular component** |
| *PMPCA* | Peptidase, mitochondrial processing subunit alpha | 0.20 | 0.52 | -1 | <0.001 | 0 | 0 | 1 |
| *SON* | SON DNA and RNA binding protein | 0.49 | 0.30 | -1 | <0.001 | 0 | 0 | 0 |
| *NDUFA9* | NADH:ubiquinone oxidoreductase subunit A9 | 0.41 | 0.20 | 1 | <0.001 | 0 | 0 | 1 |
| *SIRT5* | Sirtuin 5 | 0.44 | 0.20 | 1 | <0.001 | 0 | 2 | 1 |
| *HNRNPC* | Heterogeneous nuclear ribonucleoprotein C | 0.46 | 0.18 | -1 | <0.001 | 2 | 1 | 1 |
| *SBF1* | SET binding factor 1 | 0.49 | 0.17 | 1 | <0.001 | 0 | 0 | 0 |
| *EIF2S1* | Eukaryotic translation initiation factor 2 subunit alpha | 0.47 | 0.17 | 1 | <0.001 | 1 | 0 | 0 |
| *ZBTB38* | Zinc finger and BTB domain containing 38 | 0.46 | 0.16 | -1 | <0.001 | 1 | 0 | 0 |
| *CBX1* | Chromobox 1 | 0.30 | 0.16 | -1 | <0.001 | 1 | 2 | 2 |
| *AMT* | Aminomethyltransferase | 0.43 | 0.15 | 1 | <0.001 | 0 | 0 | 1 |
| *PRMT5* | Protein arginine methyltransferase 5 | 0.34 | 0.15 | 1 | <0.001 | 3 | 2 | 0 |
| *POLE3* | DNA polymerase epsilon 3, accessory subunit | 0.45 | 0.15 | 1 | <0.001 | 1 | 2 | 1 |
| *RRM1* | Ribonucleotide reductase catalytic subunit M1 | 0.53 | 0.15 | 1 | <0.001 | 0 | 0 | 0 |
| *NCAPH2* | Non-SMC condensin II complex subunit H2 | 0.34 | 0.15 | -1 | <0.001 | 1 | 0 | 0 |

Abbreviations: HC=healthy controls; GO=gene ontology; PMS=progressive multiple sclerosis; RRMS=relapsing-remitting multiple sclerosis.

**Supplementary Table 5: Genes showing a significant and reliable spatial association (chance likelihood <0.001 and auto-correlation ≥0.2) and an adjusted cross-correlation with a R^2^ ≥0.15 with the t value map of higher centrality in cognitively impaired MS patients compared to cognitively preserved MS patients and HC. Direction (slope) of the cross-correlations and frequencies of gene inclusion in ToppGene Suite Gene Ontology are also shown.**

| **Gene ID** | **Gene name** | **Gene**  **auto-correlation** | **Cross-correlation** | | | **ToppGene Suite GO** | | |
| --- | --- | --- | --- | --- | --- | --- | --- | --- |
|  |  |  | **R^2^-adjusted** | **Direction**  **(slope)** | **p value** | **Molecular function** | **Biological**  **process** | **Cellular component** |
| *DNASE1* | Deoxyribonuclease 1 | 0.15 | 0.35 | -1 | <0.001 | 0 | 0 | 0 |
| *CP* | Ceruloplasmin | 0.47 | 0.26 | -1 | <0.001 | 0 | 0 | 0 |

Abbreviations: HC=healthy controls; GO=gene ontology; MS=multiple sclerosis.
